# Editorial: Nutrients and bioactive compounds: preventing and treating neurodegenerative diseases and disorders

**DOI:** 10.3389/fnut.2026.1908638

**Published:** 2026-07-24

**Authors:** Daniel Ortuño-Sahagún, Lucrecia Carrera-Quintanar

**Affiliations:** 1 Laboratorio de Neuroinmunobiología Molecular, Instituto de Neurociencias Traslacionales, Centro Universitario de Ciencias de la Salud, Universidad de Guadalajara, Guadalajara, Mexico; 2 Departamento de Clínicas de la Reproducción Humana, Crecimiento y Desarrollo Infantil, Instituto de Investigación en Cáncer en la Infancia y Adolescencia, Centro Universitario de Ciencias de la Salud, Universidad de Guadalajara, Guadalajara, Mexico; 3 Departamento de Alimentación y Nutrición, Doctorado en Ciencias de la Nutrición Traslacional, Centro Universitario de Ciencias de la Salud, Universidad de Guadalajara, Guadalajara, Mexico

**Keywords:** Alzheimer disease, immunomodulation, neurodegenerative diseases (MeSH), neurodegenerative disorders, neuroinflammation, nutraceuticals, Parkinson's disease, bioactive compounds

Neurodegenerative diseases (NDs), including Alzheimer's disease (AD), Parkinson's disease (PD), and related disorders, representing a growing global health crisis driven by population aging and the lack of broadly effective disease-modifying therapies (1, 2). These conditions share core pathological features—such as oxidative stress, chronic neuroinflammation, mitochondrial dysfunction, impaired proteostasis, synaptic loss, and epigenetic dysregulation—that progressively erode neuronal integrity and cognitive function. While pharmacological options remain largely palliative, increasing evidence positions dietary nutrients and bioactive compounds as accessible, multi-target interventions capable of influencing disease onset and progression by modulating cellular and molecular pathways (3–5). The Research Topic “*Nutrients and bioactive compounds preventing and treating neurodegenerative diseases and disorders*” brings together cutting-edge preclinical, translational, and clinical insights into how specific bioactive compounds and dietary patterns can attenuate the mechanisms underlying these diseases and disorders. Bridging molecular neuroscience with nutritional strategies, it provides a timely platform for exploring prevention and adjunctive care.

This Research Topic of contributions—including original mechanistic studies, pilot trials, systematic reviews, meta-analyses, and opinion pieces—offers a coherent framework that underscores the relevance of plant-derived polyphenols, organosulfur compounds, spices, and whole-diet patterns. Across these articles, several interconnected themes emerge: antioxidant restoration, anti-inflammatory and immunomodulatory balance, mitochondrial and synaptic protection, epigenetic regulation, and nutrigenomic effects. The Research Topic consistently acknowledges key translational challenges such as bioavailability and inter-individual variability.

Antioxidant defense and redox homeostasis are foundational themes. Li et al. demonstrated that bound polyphenolic fractions from *Astragalus membranaceus* residues exhibit superior free-radical scavenging capacity and, in H_2_O_2_-challenged PC12 cells, significantly increase SOD and CAT activities while reducing ROS and MDA levels by downregulating the stress-related genes *Mmp9* and *Sesn2*. Similarly, Kara et al. showed that a standardized bilberry (*Vaccinium myrtillus*) extract rich in anthocyanins provides robust antioxidant activity across multiple assays, upregulates BDNF, and protects SH-SY5Y neurons from oxidative insult. Extending these findings *ex vivo*, Gavilan et al. found that elephant black garlic extract, enriched in unique sulfur metabolites, preserves mitochondrial membrane potential, ATP production, and Mitofusin-1 expression while restoring synaptic proteins SV2 and PSD-95 and spontaneous calcium signaling in hippocampal slices and neurons exposed to soluble Aβ. Together, these studies illustrate how sustained-release or synergistic bioactives can effectively counteract the redox imbalance central to ND progression.

Anti-inflammatory and immunomodulatory actions further strengthen neuroprotection and underscore immunomodulation as a key neuroprotective mechanism (6, 7). Bahmani et al. reported that intrathecal silymarin in a rat model of spinal cord injury improved sensorimotor recovery, preserved ventral-horn neurons, and shifted the MMP2/MMP9 balance in a favorable direction, indicating effective suppression of secondary inflammatory cascades. Alam et al. showed that curcumin-piperine nanoparticles in a cuprizone demyelination model restored hippocampal antioxidant enzymes, markedly reduced astrocyte activation and chemokine expression, and improved cognitive performance while promoting remyelination—effects superior to curcumin alone due to enhanced bioavailability. Complementing these findings, Christimann et al. synthesized evidence that components of the Mediterranean and MIND diets, including polyphenols and fiber-derived short-chain fatty acids, attenuate NF-κB signaling and microglial activation via the gut–brain axis, providing mechanistic coherence for the observed epidemiological risk reduction.

Several studies support mitochondrial resilience, synaptic integrity, and cognitive outcomes. Balbuena et al. used laser-capture transcriptomics to show that dietary curcumin in mice fed a high-glycemic diet reverses hundreds of neurodegeneration-associated genes in hippocampal microvessels, downregulating oxidative phosphorylation components, APP processing, and tau kinases while upregulating protective miRNAs and BDNF-related signaling. Gavilan et al. further linked mitochondrial stabilization to preserved BDNF levels and neuronal arborization. Clinically, Ito et al., in a 12-week randomized, double-blind, placebo-controlled pilot study, found that daily cumin essential oil significantly improved psychomotor speed and reaction time in healthy elderly adults—domains particularly vulnerable in early neurodegeneration—and this was supported by *in vitro* evidence of protection against Aβ42-induced cytotoxicity. Reinforcing these findings, Victoria-Montesinos et al. systematic review and meta-analysis of 18 RCTs confirmed that *Zingiberaceae*-derived interventions yield statistically significant improvements in memory-related outcomes, with mechanistic underpinnings involving Nrf2 activation and reduced oxidative stress, although effects on other cognitive domains remain heterogeneous.

Epigenetic and nutrigenomic regulation provide a deeper important layer. Silva-Martínez et al. reviewed how one-carbon nutrients (folate, vitamin B12, choline, and SAM) and specific bioactives (EGCG, omega-3 PUFAs, Dendrobium alkaloids, and Ganoderma extracts) modulate DNA methylation at the APP promoter, repress amyloidogenic processing, and reduce Aβ accumulation in preclinical AD models. Balbuena et al. and Singh et al. broaden this perspective to nutrigenomic and precision nutrition frameworks, showing that curcumin and related compounds influence non-coding RNAs, transcription factor networks, and individualized metabolic profiles, to enable stage-specific interventions.

Supporting these mechanistic insights, major epidemiological and clinical syntheses confirm that adherence to polyphenol-rich, plant-forward dietary patterns is associated with a 17%−30% lower risk of dementia and slower cognitive decline, while emphasizing the need for improved bioavailable formulations and longer-term trials (8, 9).

**Figure 1:**
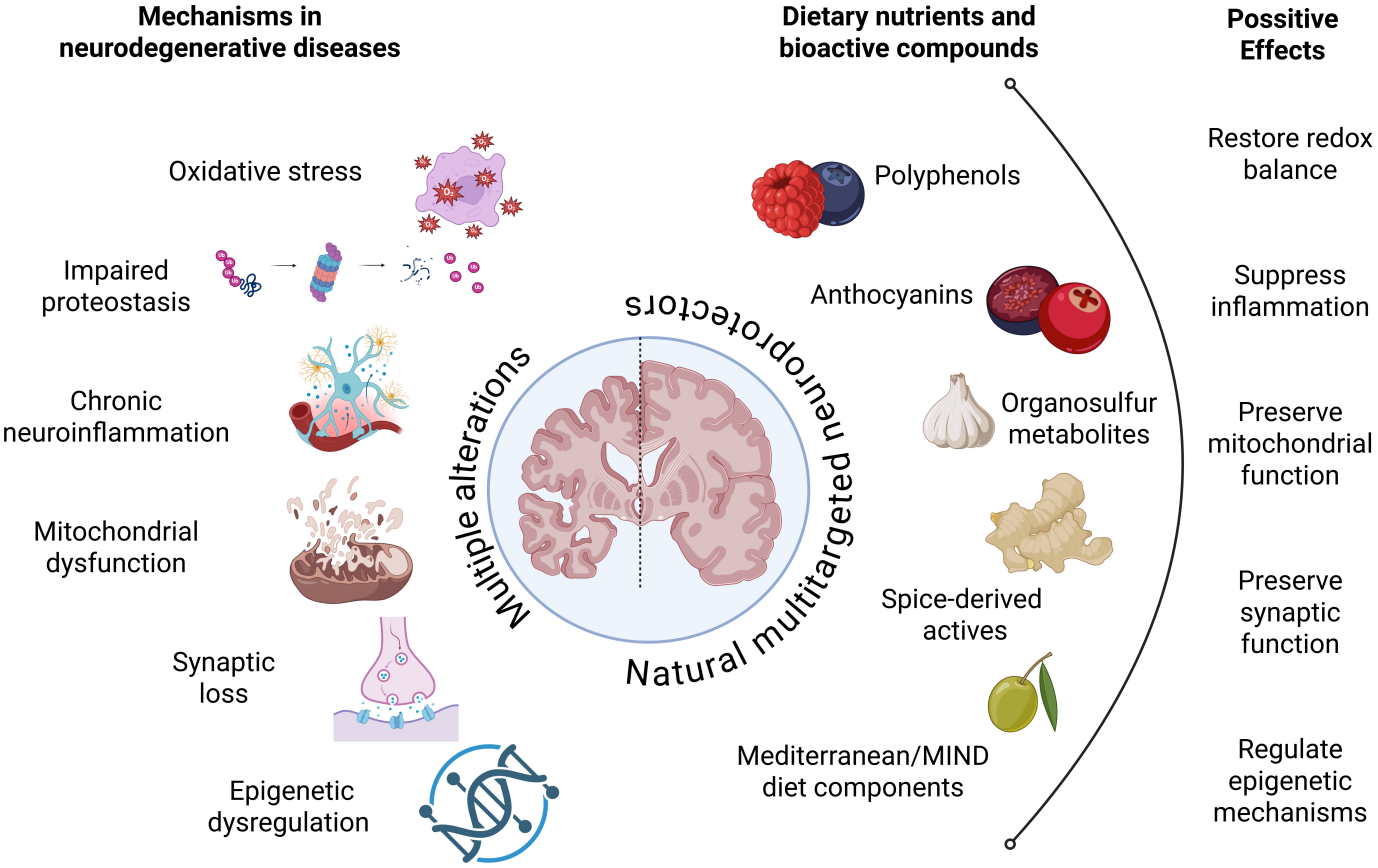
Nutrients and bioactive compounds: preventing and treating neurodegenerative diseases and disorders. Neurodegenerative diseases are driven by multiple interconnected pathological processes, including oxidative stress, chronic neuroinflammation, mitochondrial dysfunction, impaired proteostasis, synaptic loss, and epigenetic dysregulation (left side). Dietary nutrients and bioactive compounds—such as polyphenols, anthocyanins, organosulfur metabolites, spice-derived actives, and Mediterranean/MIND diet components—act as multi-target modulators that simultaneously restore redox balance, suppress inflammation, preserve mitochondrial and synaptic function, and regulate epigenetic mechanisms (right side), thereby shifting disease trajectories toward neuroprotection, prevention from midlife, and adjunctive therapeutic strategies. Created with BioRender.com.

The articles in this Research Topic converge on several key conclusions (Figure 1). First, multi-target bioactives, particularly bound or formulated polyphenols (Li et al., Kara et al., Alam et al.), organosulfur compounds (Gavilan et al.), and spice-derived actives (Ito et al., Victoria-Montesinos et al.)—can simultaneously address oxidative stress, inflammation, mitochondrial impairment, and synaptic dysfunction at cellular, tissue, and whole-organism levels. Second, dietary patterns such as the Mediterranean and MIND diets offer sustainable, synergistic advantages over single-nutrient or restrictive approaches (Christimann et al.), with substantial mechanistic overlap in anti-inflammatory and metabolic pathways. Third, epigenetic and nutrigenomic modulation (Silva-Martínez et al., Balbuena et al.) and precision nutrition frameworks (Singh et al.) represent promising avenues for personalization, although bioavailability barriers, disease-stage specificity, and translation gaps remain significant challenges. Fourth, preliminary human data from pilot RCTs and meta-analyses are encouraging but require larger, well-powered trials with standardized endpoints, biomarker stratification, and extended follow-up.

Collectively, this Research Topic advances nutritional neuroscience by providing rigorous, evidence-based demonstrations that nutrients and bioactive compounds can meaningfully modify neurodegenerative trajectories. While not a substitute for pharmacological innovation, these findings strongly support integrating targeted dietary strategies, leveraging enhanced formulations, synergistic combinations, and precision approaches, into prevention paradigms from midlife onward and as adjunctive therapies. Future efforts should prioritize *in vivo* validation, sex-specific responses, gut–brain–microbiome interactions, and pragmatic clinical trials to translate these molecular insights into tangible public-health impact. By illuminating both the promise and the remaining challenges, the contributions in this Research Topic chart a clear path toward nutrition-informed strategies that may meaningfully help alleviate the growing burden of NDs.
